# Supplementary material for: Are carbon emissions trading and green financial instruments synergistic? -Comprehensive quantitative research based on content analysis
Source: PLoS One. 2024 Mar 7;19(3):e0298601. doi: 10.1371/journal.pone.0298601 (PMC10919601; doi:10.1371/journal.pone.0298601)
Supplement: S1 Data — (ZIP) [file pone.0298601.s001.zip › Data/地级市Z_F工作报告.zip!/╣π╢1⁄2/╣π╓▌╩╨/└┤╫╘╣1⁄2╓┌║┼í╛┬φ┐╦╩2╛▌═°í┐_2016.docx]

各位代表：

　　我代表市人民政府向大会作政府工作报告，请予审议，并请政协委员和其他列席人员提出意见。

　　一、“十二五”时期经济社会发展回顾

　　“十二五”时期，是广州成功举办亚运、亚残运之后增创新辉煌的五年。面对错综复杂的国内外环境和繁重的改革发展稳定任务，我们在党中央、国务院和省委、省政府及市委的正确领导下，适应经济发展新常态，坚持为民用心做事，全力以赴稳增长、促改革、调结构、惠民生、增后劲，圆满完成了“十二五”时期目标任务，国家中心城市地位进一步巩固提升。

　　——经济实力跃上新台阶。5年来，经济保持快速增长，去年地区生产总值1.81万亿元，比上年增长8.4%，较2010年净增7352亿元，连续27年位居国内城市第3位。人均生产总值突破2万美元，实现了新的跨越。经济质量效益显著提升，去年地方一般公共预算收入1349.1亿元、比上年增长8.5%，有效防范化解财政和金融风险，财政透明度位居全国前列。产业结构明显优化，形成9个千亿级产业集群，认定总部企业320家，服务业增加值突破1万亿元，三次产业比重为1.26︰31.97︰66.77。

　　——人民生活明显改善。5年来，政府财政投入民生社会事业8813亿元，是“十一五”时期的2.4倍。城乡常住居民可支配收入明显增长，预计去年分别达4.66万元、1.93万元，年均增速分别为10.8%、12.4%。5年新增城镇就业人数211.54万人，成为全国创业先进城市。社会保障水平全面提升，城乡居民基本养老保险金人均增长89.41%，新增和筹集保障性住房16.68万套，新增管道燃气用户110.8万户、覆盖率56%。公共服务设施进一步完善，广州文化馆、美术馆开工建设，广州图书馆新馆、13个儿童公园建成开放。教育文化卫生事业全面进步，所有区成为“全国义务教育发展基本均衡县”，《广州大典》一期编纂出版，人均期望寿命等居民健康指标接近发达国家水平。民生发展指数连续两年全国城市排名第一。

　　——城乡面貌有新变化。5年来，巩固提升亚运成果，“天更蓝、水更清、路更畅、房更靓、城更美”取得新成效。空气质量持续改善，去年达标天数312天，比上年增加30天，PM2.5平均浓度降至39微克/立方米，下降20.4%。水环境治理取得进展，珠江广州河段保持Ⅳ类，城市集中式饮用水源地水质达标率达100%。破解垃圾围城难题，6个资源热力电厂全面建设，成功创建全国首批生活垃圾分类示范城市。绿化美化水平持续提升，新建绿道3000公里，海珠湿地、南沙湿地、花都湖等公园建成开放，森林覆盖率达42%。旧城面貌发生新变化，同德围、金沙洲、金融城、琶洲西区、广钢新城、广纸片区等成片连片城市更新项目稳步推进。市民对城市形象满意率比2010年提升16个百分点。

　　——国家中心城市功能不断强化。5年来，空港、海港、铁路港、信息港、城市路网等一大批重大基础设施建设全面提速。机场旅客吞吐量5520.94万人次；港口货物和集装箱吞吐量分别为5.21亿吨、1762万标箱；广珠、广深客运专线和贵广、南广高铁相继开通；高速公路通车972公里，地铁通车9条266公里；光纤覆盖用户超过700万户、入户率62.3%，国家超算广州中心天河二号运算速度全球6连冠。城乡水、电、气等民生及科教文卫政法养老基础设施建设扎实推进。

　　——城市活力和影响力大幅提升。5年来，保持改革开放先行先试优势，推出系列改革举措，南沙新区、南沙自贸试验区、珠三角国家自主创新示范区、国家知识产权示范城市获批，国家新型城镇化、旅游综合改革等试点全面推进，4次荣登福布斯中国大陆最佳商业城市榜首。对外开放取得新进展，创设广州国际城市创新奖，当选世界城市和地方政府联盟、大都会协会联合主席城市，国际友好城市和友好合作城市增至63个，3次评为“魅力中国”外籍人才眼中最具吸引力的城市。承办亚信2015年首次特别工作组和高官委员会会议，成功举办广州马拉松赛、世界羽毛球锦标赛等国际赛事，职业足球夺得2次亚冠联赛、1次足协杯、5次中超冠军。

　　过去5年，是我市经济社会发生重大变化的5年，我们迎难而上，积极作为，主要做了以下工作：

　　（一）稳增长调结构，促进经济提质增效。面对“三期叠加”的严峻形势，坚持把握发展主线，全力稳定经济增长，不断优化产业结构。

　　以重大项目建设带动发展。发挥投资的关键作用，突出重大项目带动功能，5年安排429个市重点建设项目、完成投资超4200亿元，拉动全社会固定资产投资2.19万亿元，年均增长12.7%，去年总投资5405.95亿元，是2010年的1.66倍。连续开展系列招商引资引智引技活动，吸引乐金显示、北汽、中国移动、中国联通、阿里巴巴、腾讯、国美、小米等一大批高科技项目、优质品牌和总部企业进驻。推进南沙自贸试验区、金融城、琶洲互联网创新集聚区、中新广州知识城等重大平台建设，形成一批功能突出、特色鲜明、布局合理、支撑全市发展的平台。

　　以扩大和提升有效需求促进增长。持续开展国际购物节、美食节等促销活动，推动传统专业批发市场和重点商圈升级改造，开展系列商贸改革试点，发展电子商务，培育信息、旅游休闲、健康养老等新兴消费，千年商都地位巩固提升。去年全社会消费品零售总额7933亿元、比上年增长11%，居全国第3位。全力提振外贸转型升级，出台稳定外需政策措施，发展外贸新业态，打造国家电子商务示范城市、跨境电商服务试点等14个开放型经济平台，增强了外需拉动作用。去年全市进出口总额1338.7亿美元，比上年增长2.5%，其中出口增长11.6%；跨境电商进出口67.5亿元，增长3.7倍，居全国第一。5年累计实际使用外资241.8亿美元，境外投资100.51亿美元，分别是“十一五”时期的1.4倍和6.2倍。

　　以创新推动产业转型升级。在传统增长动力减弱的情况下，加大科技创新力度，出台“1+9”科技创新系列政策，实施财政科技投入和孵化器双倍增计划，推进知识产权示范和枢纽城市建设，支持设立广州知识产权法院，加强知识产权保护，科技创新引领作用不断增强。去年财政科技支出71.7亿元，是2010年的4.68倍，科技企业孵化器119家、孵化面积650万平方米，分别是2010年的4.25倍和3.76倍，在孵企业超过6000家，工业高新技术产品产值8420.56亿元，发明专利授权6626件、比上年增长44.4%，企业专利质押融资1.34亿元。加快现代服务业发展，深化国家服务业综合改革试点，建设一批省级现代服务业集聚区，大力发展总部经济、现代金融、现代物流，积极培育卫星导航、科技服务、工业设计、文化创意等新业态，现代服务业增加值占服务业比重63.5%，金融业增加值占地区生产总值比重提升至9%。大力发展先进制造业，完成“退二”企业314家，推动600多家规模以上工业企业新一轮技术改造，打造智能装备及机器人、船舶及海洋工程装备、核电装备等先进制造业基地，建设中小企业先进制造业中外合作区，组建智能装备研究院，壮大新一代信息技术、生物医药等战略性新兴产业，推动制造企业应用电子商务开拓市场。去年规模以上工业总产值1.87万亿元，按可比价格是2010年的1.63倍；预计战略性新兴产业增加值比上年增长10.2%。大力发展现代农业，建设39.67万亩高标准基本农田、4.5万亩标准化菜田和一批“菜篮子”生产基地，初步建成市级现代农业园区8个，农业龙头企业增至83家，农民专业合作社1151家。

　　（二）深化改革开放，激发市场活力。针对束缚发展的体制机制障碍，加大力度推动改革落地，为发展提供强劲动力。

　　推进重点领域改革。深化行政体制改革，完成增城、从化、黄埔行政区划调整，推进政府职能转变和机构改革，公布权责清单；加大简政放权力度，动态调整行政审批备案事项641项，全部取消非行政许可审批事项，优化审批流程，推行国际贸易“单一窗口”和口岸管理“三互”机制，探索企业投资“三单”管理。深化商事制度改革，推行工商登记“三证合一、一照一码”，推进市场监管体系和社会信用体系建设，加强事中事后监管，5年新增商事主体79万户、注册资本总额1.4万亿元。深化财税改革，建立全口径政府预决算编报体系，率先全国公开三级政府“三公”经费及会议费；落实“营改增”和小微企业税费优惠政策，停征堤围防护费和停征或取消行政事业性收费70项、5年为企业减负65亿元。加快投融资体制改革，创建民间金融街等6大金融功能区，设立广州碳排放权交易所等5大金融交易平台，组建广州基金投融资平台，上市企业115家，新三板挂牌企业146家，广州股权交易中心挂牌企业2994家。深化国有企业改革，实现市属经营性国有资产集中统一监管，筹建国有资本运营公司，推动上市公司二次混改，完善公司法人治理结构，推进国企兼并重组，5年累计上缴收益和税费2300多亿元。深化价格改革，实行居民用户阶梯水价和气价制度。深化社会管理改革，铺开社会组织直接登记，在荔湾区开展“一窗式”政务服务改革试点，创建一卡、一号、一格、一网、一窗“五个一”社会治理模式。深化农村综合改革，推进集体建设用地使用权流转，建成集体经济“三资”交易管理平台，启动农村土地承包经营权确权工作，农村集体土地所有权、宅基地使用权、集体建设用地使用权确权登记发证率达99.6%以上。

　　加快南沙新区和南沙自贸试验区建设。把南沙自贸试验区建设作为改革开放的试验田，带动南沙新区和全市创新发展。创新制度机制，推行“一口受理”新模式，实施负面清单管理，推动穗港澳服务贸易自由化，组建国际航运仲裁院，首创“智检口岸”模式和政府购买查验服务，海关通关时效提高50%以上。明确功能片区定位，铺开基础设施建设，总投资1557亿元，累计新设企业7589家。开展汽车平行进口，建立跨境商品直购体验中心，706家跨境电商企业完成检验检疫备案，初步形成经济增长新引擎。

　　深化交流合作。创建广州－奥克兰－洛杉矶三城经济联盟，贸易往来国家和地区200多个。积极参加国际区域合作，成功举办2015广东21世纪海上丝绸之路国际博览会和第九届中国企业跨国投资研讨会。推进区域协同发展，参与珠江－西江经济带合作，泛珠合作论坛暨经贸洽谈会永久会址落户广州，加强与周边六市跨区域交通基础设施建设，推进广佛同城化、广清一体化及广佛肇清云韶经济圈建设，推进梅州、清远、湛江帮扶，对口支援新疆疏附、西藏波密、贵州黔南等地区成效明显。

　　（三）建设宜居宜业城市，提升城市综合承载力。始终坚持建设国家中心城市目标不动摇，着力提升城市核心枢纽功能，不断增强综合竞争力和国际影响力。

　　规划引领城市发展。编制城市总体规划和“一江两岸三带”、重大发展平台、江心岛、都市区生态轴廊道、历史文化街区保护等专项规划，率先在全国副省级城市实施“三规合一”，实现国土、规划和经济空间“一张图”动态管理，盘活用地128平方公里。推进37宗“烂尾楼”盘活工作。开展城乡统筹土地管理创新试点，5年新增土地红线储备253平方公里、实物储备结案面积77平方公里，供应建设用地90平方公里。完成889条行政村规划，创建一批国家级、省级名镇名村，建设89个美丽乡村。

　　加强基础设施建设。加快白云机场扩建、南沙港三期、国铁、城轨、地铁、高快速路和市政路桥等一批重大项目建设，白云机场第三跑道投入运行，新增13条国际航线，机场噪音区治理稳步推进；广州港出海航道三期建成，新增外贸班轮航线15条，新建10个内陆无水港或办事处，广州港深水航道拓宽工程、南沙港四期、南沙国际邮轮码头建设加紧推进；新建成311公里高速公路和234公里市政道路桥梁，开通海珠新型有轨电车试验线，开工建设3个国铁项目、5个城际轨道项目，推进11条地铁建设，新增公交专用道375公里,优化14条水上巴士线路。大学城提升工程、教育城一期、5个医疗机构项目建设取得进展，动工建设9个公办养老机构，其中5个完工，38个政法基础设施加紧建设，城市功能进一步完善。

　　优化城乡生态环境。落实“天更蓝”八大措施，完成21台煤电机组“超洁净排放”改造、总装机容量463万千瓦，淘汰高污染燃料锅炉1298台、黄标车18万辆，4个区建成“无燃煤区”，实现机动车国Ⅳ排放标准全覆盖，强化工地道路扬尘、挥发性有机物、餐饮业油烟排放控制，推广新能源汽车，推动1282家企业开展清洁生产。实施“南粤水更清”行动，推进51条河涌整治，建成污水处理厂5座，在建14座，新增污水处理能力34万吨/日，城乡生活污水处理率分别达92.5%、48%；完成城乡自来水厂达标改造，加强流溪河饮用水源保护。逐步推广生活垃圾定时定点分类投放，完善垃圾分类收运配套建设，完成91座垃圾压缩站分类收运升级改造和36个“一镇一站”建设，加快建设垃圾终端处理设施。加强重金属、农药残留和固体废弃物等土地面源污染治理。推进新一轮“绿化广东”大行动和花城绿城建设，建设森林公园和湿地公园，实施碳汇造林，建设生态景观林带，加强乡村绿化美化，打造北部旅游、中部休闲、南部滨水生态片区。

　　强化城市综合治理。加强城管综合执法，提高市容景观管理水平，集中整治“五类车”、乱摆卖和占道经营，5年累计拆除违法建设600多万平方米、违法户外广告30万平方米，查处违法施工2.5万宗，完成151条城中村安全隐患和826个专业市场管线整治。创建全国质量强市示范城市，制造业产品质量合格率达94.68%，大中型工程一次验收合格率达100%。推进平安广州建设，坚持安全发展理念，加强安全生产监管，实施安全生产综合治理，强化城市安全与应急管理及防灾救灾能力建设，保持“打非治违”高压态势，安全生产形势平稳有序；推进“食得放心”城市建设，持续开展打击假冒伪劣专项行动，破获食品药品环保类刑事案件998宗。组建反恐力量和反电信诈骗中心，组织涉毒涉黑等“3+2”等专项打击行动，累计建成视频监控点55.9万个，完善立体化社会治安防控体系，去年刑事、治安类警情下降5.9%，群众治安满意度持续提高。推进社会治理创新，97.9%的市级行政审批事项和99.6%的社会服务事项可在网上办理，170个街镇建成“一队三中心”，50%的社区建成幸福社区，推广“民主商议、一事一议”村民自治模式，实现“一村（社区）一法律顾问”，健全村（居）务公开。加强来穗人员服务管理，开展出租屋专项治理，整治“群租房”2.66万套，完成1.65万人积分入户。健全应急管理体系，妥善处置各类突发事件，社会大局和谐稳定。

　　（四）推动社会事业发展，促进基本公共服务均等化。着力缩小地区间、城乡间、群体间公共服务差距，让发展成果更加均衡全面地惠及全体市民。

　　发展教育事业。7个区成功创建省推进教育现代化先进区，完成第一期学前教育三年行动计划，公办和普惠性民办幼儿园占比达72.8%。推动中小学教育质量综合评价改革，普通高考上线率97%。广州大学和广州医科大学全部专业纳入一本招生，构建现代职业教育体系，推动民办教育规范特色发展，特殊教育向学前教育和中职教育延伸。

　　发展文化事业。培育和践行社会主义核心价值观，推进文明城市创建活动。举办音乐金钟奖、戏剧梅花奖、国际漫画节、国际纪录片节、国际粤剧节等活动，举办中国图书馆年会，完成全市文化遗产普查，保护修缮文物建筑109宗，打造《西关小姐》等一批文化名片，做大做强游戏动漫、时装设计等文化创意产业，世界文化名城建设扎实推进。

　　发展医疗卫生事业。扩大优质医疗资源，全市三级甲等医院增至34家。深化县级公立医院改革，实施基层中医药服务能力提升工程，开展家庭医生签约服务，组建21家医联体试点，推行基层首诊、双向转诊，加强住院医师和全科医师培训，鼓励支持社会资本办医，做好埃博拉、H7N9型禽流感、登革热等传染性疾病防控和应急救治。

　　发展体育事业。推进公共体育场馆免费或低收费进场，人均体育场地面积增至2.38平方米。率先全国完成地方足协改革，举办134项国际国家级赛事，广泛开展全民健身活动，大力发展竞技体育，获得2项奥运冠军、58项世界冠军，第七届全国城运会和首届全国青运会获金牌数、奖牌数和总分第一名。

　　其他事业取得新进展。推进“双拥”和优抚安置工作，第7次荣获全国“双拥模范城”称号。完成经济普查和“六五”普法，法律援助、社区矫正、人民调解工作进一步加强。完善少数民族服务管理体系，推进民族宗教设施建设。农村扶贫开发取得成效。国防动员、海防和打私、妇女儿童、人口计生、残疾人、侨务、对台、贸促、口岸、应急、气象、地震、民防、审计、统计、参事、文史、档案、保密、修志、仲裁、红十字会等工作取得进步。

　　（五）办好民生实事，提高人民幸福感。坚持以人为本，持续增加民生投入，保基本、兜底线、建机制。每年办十件民生实事，市本级财政5年累计投入399.7亿元，解决了一批关系群众切身利益的问题，去年十件民生实事全部兑现。坚持创业带动就业，去年扶持创业2.67万人。社会保险五险缴费人数2840.73万人次,比2010年增长58.46%。企业离退休人员月均养老金3200元，农转居人员月均养老金869元，分别比2010年增长43.56%和53.26%。完善城乡居民医疗保险制度，实施城乡居民大病医疗保险，城镇职工医保和城乡居民医保统筹基金年度最高支付限额，分别达到66.82万元和30.28万元。连续9年提高城乡低保标准，建成覆盖全体居民的医疗救助体系，5年新建1142个农村老年人活动站点、170个日间托老机构，新增养老床位2.5万张、比2010年增长87%。

　　大力加强政府自身建设。推进依法行政，开展法治示范单位创建活动，5年配合市人大完成地方性法规草案30项，提交市人大常委会审议通过23项，出台政府规章82项、政府规范性文件182项，受理行政复议案件16920宗,推行政府法律顾问制度，法治政府建设位居全国前列。推进阳光政府建设，实施重大行政决策事项目录、听证目录和专家论证办法，实行重大民生决策公众咨询监督制度，首创市政府领导定期、市政府常务会议后即时新闻发布制度。加强廉政建设，严格落实中央八项规定精神，开展公共服务领域廉洁化活动，清理行政事业单位“小金库”，持之以恒查纠“四风”，推进市管国企巡察全覆盖，加强行政监察，推进行政权力公开运行，加强政府重大投资项目廉情预警评估及廉政风险防控，成功整合公共资源交易五大平台。自觉接受人大监督，定期向市人大报告工作、向市政协通报工作，5年办理全国政协委员提案7件、市人大议案决议5件、省市人大代表建议1801件、省市政协委员提案2649件。去年的932件各级人大代表建议、政协委员提案全部办结。

　　回顾过去5年，我们深切体会到，做好政府工作，必须始终坚持党的领导，以“四个全面”战略布局为统揽，保持战略定力，推动科学发展和可持续发展；必须紧紧依靠全市人民，激发创新活力，共同面对棘手问题，勇于破解发展难题；必须牢记坚持为民用心做事的施政理念，把为人民谋发展增福祉作为最大责任，坚持问政于民、问计于民、问需于民、问效于民，扎扎实实为群众办实事、办好事；必须认真抓好政府自身建设，依法行政，阳光行政，注重廉洁高效，彰显社会公平正义。

　　各位代表，过去5年，成绩来之不易。这是党中央、国务院和省委、省政府及市委正确领导的结果，是市人大、市政协大力支持和有效监督的结果，是全市人民团结奋斗的结果。我代表广州市人民政府，向全市人民、驻穗部队官兵、各民主党派、人民团体以及社会各界人士表示崇高的敬意！向关心支持广州改革发展的港澳台同胞、海外侨胞、国际友人表示衷心的感谢！

　　总结过去，我们清醒地看到，我市经济社会发展中还存在不少问题。主要是：经济增长压力较大，工业投资增速较慢，各区产业结构趋于同质化，产业集聚集群集约程度不高，现代服务业比重相对较低，先进制造业新增长点不多，战略性新兴产业、新业态尚未形成有效支撑，总部经济发展与中心城市地位不相适应。科技创新能力不足，资源整合利用不够，社会创新意识不浓，高端人才和创新型领军企业不多，成果转化率不高。城市环境还有待改善，生态环境污染压力增大，农村污水处理率较低，河涌治理进度较慢，城市交通拥堵问题比较严重，城市治理精细化品质化水平不高，部分城中村、出租屋脏乱差现象仍然突出，城市安全与应急管理及防灾救灾能力建设有待加强。政府职能转变还不到位,市场化国际化法治化营商环境仍需优化。民生社会事业还有不少薄弱环节，城乡一体化发展和扶贫开发工作任重道远，基本公共服务均等化水平不高，来穗人员服务管理有待加强，就业、社保、住房、教育、医疗、食品安全、水电气等民生社会事业与群众期望还有差距。政府系统党风廉政建设和反腐败斗争形势依然严峻复杂，一些领域腐败问题仍然易发多发。对此，我们必须高度重视，勇于担当，努力加以解决。

　　二、“十三五”时期的主要目标和任务

　　根据《中共广州市委关于制定国民经济和社会发展第十三个五年规划的建议》，我们编制了《广州市国民经济和社会发展第十三个五年规划纲要（草案）》，提交大会审议。

　　“十三五”时期是全面建成小康社会的决胜阶段。世界经济在深度调整中曲折复苏，进入国际贸易规则重塑和产业转型发展期。国内经济发展进入新常态，经济发展方式加快转变，特别是国家深入实施“一带一路”、自由贸易试验区、“中国制造2025”、“互联网+”、大数据等重大战略，为经济发展注入新动力。我省深入实施创新驱动战略，建设自主创新示范区，促进珠三角地区优化发展和粤东西北地区振兴发展，为强化国家中心城市带动作用提出更高要求。我市千年商都底蕴深厚，科教文卫体资源集中，环境宜居宜业，敢为人先、务实进取的干事创业氛围浓厚，发展态势蓬勃向上。综合判断国内外形势，我市发展正处于大有作为的重要战略机遇期，也面临诸多挑战。我们必须保持清醒头脑，增强忧患意识，努力推动政府工作再上一个新台阶。

　　今后5年政府工作的总体要求是：高举中国特色社会主义伟大旗帜，全面贯彻党的十八大和十八届三中、四中、五中全会精神，以马克思列宁主义、毛泽东思想、邓小平理论、“三个代表”重要思想和科学发展观为指导，深入贯彻习近平总书记系列重要讲话精神，以“四个全面”战略布局为统揽，坚持发展是第一要务，坚持以人为本和可持续发展，牢固树立创新、协调、绿色、开放、共享的发展理念，落实省委、省政府和市委部署要求，以提高发展质量和效益为中心，加快形成引领经济发展新常态的体制机制和发展方式，坚持稳中求进，突出价值创新，实施创新驱动发展核心战略，加快形成新的发展动力源和增长极，优化城市空间布局，推进智慧城市建设，加快产业转型升级，建设国际航运中心、物流中心、贸易中心、现代金融服务体系和国家创新中心城市，打造国际航运枢纽、国际航空枢纽、国际科技创新枢纽，营造市场化国际化法治化营商环境和干净整洁平安有序城市环境，促进投资贸易便利化和生活服务便利化，发展高水平开放型经济，促进文化大发展大繁荣，建设绿色生态美丽广州，维护社会和谐稳定，增进民生福祉，推动广州国家中心城市建设全面上水平，在率先全面建成小康社会、率先基本实现社会主义现代化的实践中走在前列。

　　今后5年经济社会发展的主要目标是：在确保率先全面建成小康社会的基础上，着力强化国家中心城市功能，经济发展提质增效，创新能力显著增强，改革开放走在前列，城市环境宜居宜业，城乡一体协调发展，民生福祉持续改善，文明程度明显提高。到2020年，地区生产总值达到2.8万亿元，年均增长7.5%以上，人均生产总值达到18万元左右，城乡居民收入与经济发展同步增长，提前实现全市生产总值和城乡居民人均收入比2010年翻一番。

　　实现上述目标，必须认真贯彻市委十届七次、八次全会精神，着力在以下几个方面狠抓工作落实：

　　——着力推动形成新的动力源和增长极。以价值创新为引领，聚焦“三中心一体系”建设，重点建设“三大战略枢纽”，优化提升“一江两岸三带”，形成“多点支撑”格局，优化区域生产力布局和产业链对接，拓展发展新空间。在适度扩大总需求的同时，推进供给侧结构性改革，实施现代服务业和先进制造业双轮驱动战略，深化旅游综合改革，发展都市型现代农业，培育战略性新兴产业和新业态，服务业增加值占地区生产总值比重达70%，构建产业新体系。推进以科技为核心的全面创新，加快建设国家创新中心城市，培育创新主体，加强创新载体建设，实施人才强市和知识产权战略，营造良好创新生态环境，推进大众创业、万众创新，全社会研发投入占地区生产总值比重达3%，推动创新驱动发展走在全国前列。大力发展高效安全的网络经济，加强信息基础设施建设，实施“互联网+”行动计划和大数据战略，提高智慧城市建设水平，抢占未来竞争制高点。

　　——着力统筹协调城乡区域一体化发展。科学布局建设综合交通运输体系，大力推进海港、空港、高速铁路、城际轨道、高快速路和地下管廊等重大基础设施建设，加快推动新一轮地铁线网、高快速路网建设，继续实施公交都市战略，优先发展城市公共交通，改善自行车、步行交通环境，建设慢行交通系统，加快中心城区周边市政道路建设，提升大交通综合枢纽地位。深入推进城市环境综合整治，加强城市容貌景观治理，加强精细化品质化管理，全面实施城市更新政策，建设干净整洁平安有序的生态宜居城市。创新社会治理，组织开展幸福社区提升计划，推进城市社区网格化服务管理，探索新型农村社区建设模式和农民生产生活社会化服务模式，推进基层民主建设，开展群防群治，深化平安广州建设。推动城乡一体化，推进新型城镇化，完善镇村基础设施，深化农村综合改革，建设美丽乡村。传承和发扬岭南文化，开展社会主义核心价值观教育，实施重大文化工程，加快文化产业转型升级，全面提升历史文化名城的软实力。

　　——着力推进资源节约和环境保护。牢固树立生态价值观，强化约束性指标管理，实行能源和水资源消耗、建设用地等总量和强度双控行动，全面增强可持续发展能力。建设森林城市，实施重点林业园林生态建设工程，扩大湿地保护面积，提升城市园林绿化景观品质，推动乡村绿化美化，全面建成绿道网络体系，森林覆盖率达42.5%。加强环境保护与治理，加大河涌、空气、土壤污染治理力度，重点加强黑臭水体治理，提高农村生活污水处理率，强化江河水源地和水源涵养区生态保护。建设海绵城市，加强防洪排涝水利设施建设。创建国家循环经济示范城市，推动企业开展清洁生产工作，推进节能减排降耗，深化垃圾分类处理，推广绿色建筑和绿色施工，发展绿色交通，倡导绿色低碳生活方式。

　　——着力构建高水平开放型经济体系。加快南沙新区、自由贸易试验区建设，全面落实广东自由贸易试验区建设实施方案，以投资准入、贸易、金融、事中事后监管、知识产权保护等制度创新为核心，推动南沙新区建设走在前列，营造市场化国际化法治化营商环境，打造新一轮对外开放大平台。建设“一带一路”战略节点城市，加快基础设施互联互通，加强产能合作和贸易往来，发挥龙头带动和主枢纽作用。推进中新广州知识城和中欧、中以合作示范区建设。深化国际交流和区域合作，继续实施友城拓展战略，加快外经贸优化升级，推动穗港澳服务贸易自由化，深化穗台交流合作，深入推进广佛同城化、广清一体化，积极构建广佛肇清云韶经济圈，提高开放合作水平。

　　——着力保障和改善民生。按照人人参与、人人尽力、人人享有的要求，创新公共服务提供方式，提高基本公共服务优质化均等化水平，城乡居民生活品质进一步提升。健全社会保障体系，实施更加积极的就业政策，城镇登记失业率控制在3.5%以内，提升中等收入人口比重，稳步提高社保待遇，构建多层次住房保障体系，加强养老服务、社会救助、残疾人保障，提高社会保障水平。发展社会事业，深化教育领域综合改革，健全多层次城乡医疗卫生服务体系，构建和谐劳动关系，加强来穗人员服务管理，实施精准扶贫、精准脱贫，落实一对夫妇可生育两个孩子政策，促进人口均衡发展。

　　三、2016年工作安排

　　今年，是实施“十三五”规划、率先全面建成小康社会决胜阶段的开局之年。经济社会发展主要预期目标是：地区生产总值增长8%左右，地方一般公共预算收入可比增长8%，固定资产投资增长12%，社会消费品零售总额增长10%左右，商品出口总值增长8%，城乡居民收入增长与经济增长基本同步，城市居民消费价格涨幅控制在3%以内,城镇登记失业率控制在3.5%以内。

　　实现上述目标，我们要牢固树立创新、协调、绿色、开放、共享发展理念，主动引领经济发展新常态，围绕国家中心城市建设，以提高发展质量和效益为中心，坚持改革开放，坚持稳中求进工作总基调，坚持稳增长、调结构、惠民生、防风险，突出价值创新引领，推进结构性改革，培育新的动力源和增长极，营造市场化国际化法治化营商环境和干净整洁平安有序城市环境，加快建设国际航运中心、物流中心、贸易中心、现代金融服务体系和国家创新中心城市，着力建设国际航运枢纽、国际航空枢纽、国际科技创新枢纽，优化提升“一江两岸三带”， 把去产能、去库存、去杠杆、降成本、补短板任务落到实处，推动广州国家中心城市建设上水平，努力实现“十三五”经济社会发展良好开局。重点抓好以下工作：

　　（一）创新推动经济行稳致远。把创新摆在发展的核心位置，坚持价值创新引领，实施创新驱动战略，发展先发优势产业，推进经济结构性改革，提升经济发展质量效益。

　　一是加快国际科技创新枢纽建设。抓紧出台建设珠三角国家自主创新示范区方案，制定实施全面创新改革三年行动计划，落实科技创新“1+9”政策，加大财政科技投入，重点发展生物医药、计算科学应用、智能制造等专业孵化器，孵化器达到120家，孵化面积达800万平方米，新增毕业企业1000家。实施科技创新小巨人企业及高新技术企业培育行动计划，建立以企业为主体的产学研技术创新联盟，鼓励发展新型研发机构，开展一批原始创新与核心技术攻关，培育有国际竞争力的创新型领军企业，推进科技、金融、产业融合发展。运用标准化手段加快创新成果产业化、市场化，提高知识产权交易量，发展科技金融，举办创新科技成果交易会，推动科研机构体制和成果处置权、收益权改革。重点建设科技创新走廊，加快国家制造业创新中心、中新国际联合研究院、清华大学珠三角研究院、中乌巴顿焊接研究院、中科院广州生物医药与健康研究院、国家超级计算广州中心、工信部电子五所总部新区、中科院广州工业技术研究院等重大创新平台建设。培育引进创新创业人才，完善人才政策，改善人才创业发展环境，在广东国际创客中心、广州科学城、广州大学城、天河智慧城、创意大道、岭南V谷、番禺节能科技园、增城低碳总部园、中大科技园、民营科技园等园区，发展一批创客空间，创建创客空间公共服务平台，鼓励设立风投基金，支持社会组织创新，推动大众创业、万众创新。

　　二是推进经济结构性改革。制定去产能、去库存、去杠杆、降成本、补短板行动方案。支持国企和龙头企业通过兼并重组化解落后过剩产能，严把环保审批、产业政策关，杜绝新增落后产能，推动食品饮料、家具制造等富余产能向外转移，巩固“退二进三”成果。加强房地产市场形势分析和研判，发展住房租赁市场，保持房地产市场健康平稳。有效防控政府性债务风险，加强跨市场跨业务的风险传递监测，防范互联网、民间借贷等非正规金融风险扩散。开展降低实体经济企业成本行动，开辟招商引资、企业落户、项目建设等领域的绿色通道，减轻企业负担。继续加大基础设施建设力度，提升科技、金融和总部经济实力，增强城市“软实力”。

　　三是拓展城市发展新空间。围绕重点建设“三大战略枢纽”、“一江两岸三带”、“多点支撑”格局的城市空间发展战略，开展“多规合一”，加快城市总体规划报批，完善土地利用总体规划，优化城市空间布局，组织交通基础设施布局和海绵城市规划研究。推进重大平台规划编制工作，重点推进南沙自贸试验区、空港经济区、大学城创新谷等重点地区规划设计。加快白鹅潭经济区、白云新城、黄埔临港经济区、南站商务区、新中轴线南段商务区等功能区建设。推进天河路商圈、北京路文化核心区优化提升和状元谷、花地河、新塘牛仔谷等电子商务区建设，打造对外贸易集聚区。

　　四是培育新兴消费热点，提升需求质量。发挥消费的基础作用，推进国内贸易流通体制改革试点，举办国际购物节、美食节等主题展销活动，利用重大节庆时点带动传统消费，发展智能、绿色、健康、安全消费，培育新兴消费热点。强化投资带动，全年安排186个重点项目、投资1056亿元；举办国际投资年会和南沙自贸试验区、中新广州知识城、天河中央商务区推介活动，利用广交会、达沃斯论坛、海上丝绸之路博览会和主题论坛等平台渠道，吸引一批成长性好、实力强的项目落户；放宽民间投资市场准入，推出一批民生公共服务和基础设施项目向社会资本开放。实施优进优出战略，扩大先进技术设备、关键零部件和大宗商品等进口，鼓励开展飞机、船舶租赁业务，发展旅游购物、跨境电商、保税物流等外贸新业态。申报服务贸易、市场采购贸易方式等国家试点，推进服务贸易和服务外包，支持民营进出口企业做大做强。加大“走出去”企业服务力度，推动国际产能和装备制造合作，鼓励企业赴境外投资。

　　（二）构建高端高质高新现代产业发展体系。深入实施现代服务业和先进制造业双轮驱动战略，加快推进农业现代化，促进产业集聚集群集约发展。

　　一是聚焦“三中心一体系”发展现代服务业。围绕打造现代金融体系，推进国际金融城、南沙现代金融服务区、广州金融创新服务区等金融功能区建设。积极争取设立创新型期货交易所、数据交易中心等金融交易平台。新设民营银行等一批法人金融机构和组织。推动政府投融资改革，支持各区设立政府投资基金。发展170家农村金融服务站，新增10家以上境内外上市公司，新三板挂牌企业快速增长。开展物流标准化试点，推进餐饮业转型，发展冷链物流、第三方物流和供应链服务产业。继续推进传统专业市场改造升级。推进一批生产服务功能区和一批工业设计中心建设。开展工业电子商务区域试点。引进一批龙头企业在穗设立总部、区域总部、结算中心、共享服务中心等，着力打造华南总部基地。建设大数据产业示范基地，推动广州超算中心大数据应用，发展信息技术咨询服务和高端信息服务外包业态。做强广州航运交易所，探索设立珠江航运指数，发展金融保险、融资租赁、法律仲裁等现代航运服务业。推进旅游综合改革政策落地，发展健康、养老、文化、体育等新兴产业，培育新的经济增长点。

　　二是做大做强先进制造业。发展高端制造业，实施《广州制造2025战略规划》，重点培育发展智能装备及机器人、新一代信息技术、新能源汽车、生物医药和健康、新材料与精细化工、轨道交通、能源及环保、高端船舶与海洋工程装备、航空与卫星应用、都市消费工业十大重点领域,着力打造一批千亿级产业集群。发展战略性新兴产业和新业态，重点发展无线射频识别芯片、智能传感器、传感网络设备等物联网设备制造业，发展光产业，建设一批先进制造业集聚区和战略性新兴产业基地。启动一批重大技改升级工程，推进汽车制造、电子信息、纺织服装等行业智能化改造。支持新材料、汽车、船舶、医药、食品等领域建设数字化车间及智能工厂，提升传统工业生产工艺和质量水平。启动工业大数据示范应用试点，支持企业应用大数据开展生产、管理、商业模式创新。

　　三是积极发展现代农业。加强社会主义新农村建设，培育现代农业经营主体，加大力度建设高标准基本农田，加强现代农业园区、“菜篮子”基地建设，发展观光休闲等都市型农业，推进农业精细化、信息化和品牌化。推进农村土地承包经营权确权登记颁证。加强农业面源污染治理。加快推进牲畜屠宰和肉品流通经营体制改革，加强现代化屠宰场规划建设，完善农产品质量安全监管体系和动植物疫病防控体系，开展市外定点供穗农产品生产基地建设与认定。实施精准扶贫、精准脱贫，全面完成新一轮农村扶贫开发各项任务。编制海洋经济发展规划，发展远洋捕捞和养殖，建设国家海洋高技术产业基地和科技兴海产业示范基地。

　　（三）营造市场化国际化法治化营商环境。坚持深化改革、扩大开放，把广州打造成开放、包容又充满活力的城市。

　　一是推动关键领域改革落地。继续简政放权，动态调整行政审批事项，优化项目审批服务，加强事中事后监管。推进综合行政执法体系改革。进一步放宽商事主体住所、经营场所条件，推进工商登记全程电子化。推进市场监管体系和社会信用体系建设。建立市区事权和支出责任相匹配的财政体制，加大一般性转移支付力度，强化财政专项资金管理，提高预算绩效。深化“营改增”和国地税征管体制改革。推动民间资本进入金融领域，发展普惠金融，规范发展互联网金融。推动国有资本运营公司有效运作，实施“清单管理”国资监管模式，继续推进国有资产清产核资，筹建国资产业发展并购基金，推进职业经理人制度建设试点，推动公益类企业转型发展。推进国有林场和电力体制改革，组建广州电力交易中心。

　　二是推进南沙等功能区改革创新。推进南沙新区和南沙自贸试验区建设，在大通关体系、行政审批、市场准入和监管、法治环境、港澳合作模式等方面加强创新，形成一批可复制推广的改革经验；建设国际大宗商品交易中心，发展以航运金融、跨境人民币业务为重点的特色金融，推动期货保税交割仓政策落地；开展出口采购集拼，发展汽车进口和保税中转业务。创新国家新区和国家级开发区管理体制，推进广州开发区中欧区域政策合作试点和中以高技术产业合作重点区域建设，扩大增城开发区管理权限，推动中新广州知识城纳入国家战略，推进国家侨梦苑建设。

　　三是发展高水平开放与合作。围绕“一带一路”战略，推进中小企业先进制造业中外合作区建设，加快中欧区域城市合作试点，参与沿线国家基础设施建设，参与中国—东盟自由贸易区升级版建设，探索设立丝路发展基金。继续推进“三互”通关改革和国际贸易“单一窗口”建设，完善准入前国民待遇加负面清单管理模式。推进穗港澳深度融合，推动穗台交流合作，加强与高铁沿线城市的交流合作，重点推进城际轨道、高速公路、经济合作区、跨界河涌整治等重大合作项目，带动粤东西北振兴发展。推进与国际城市组织合作，举办第三届“广州奖”。做好对口帮扶梅州、清远和援疆援藏援黔工作。

　　（四）优化中心城市功能品质。围绕巩固提升国家中心城市影响力和辐射力，尊重城市发展规律，统筹城市科学发展，建设宜居宜业、绿色发展的现代化大都市。

　　一是加快国际航运枢纽和国际航空枢纽建设。加快推进白云机场二期扩建、6个港航项目、4个国铁工程、7条城际轨道、11条地铁、8条高快速公路和一大批市政工程项目建设，完善交通路网建设，打通断头路。建设一批公交设施，抓好与周边6市互联互通交通项目建设。加速国际航运、国际航空服务要素集聚，吸引国际航运服务机构落户。改革空港经济区发展体制机制，建立完善航空产业发展政策体系，推动空港经济区上升为国家航空经济示范区，建设国家跨境电商综合试验区，推进中澳新韩自贸产业园建设。推进港铁、空铁联运交通综合枢纽建设，提升交通枢纽功能。

　　二是优化提升“一江两岸三带”。加快建设精品珠江，重点打造3段10公里珠江岸线，突出一桥一风景、一桥一特色，优化提升桥梁景观，完成临江大道生态景观带、广州塔·珠江黄金水段5A旅游景区等重点项目，打造具有岭南特色的水陆双线景观。加快建设黄金三角区，推进琶洲大道、地下空间、电力电信基础设施建设，推动一批意向投资企业落户建设，推进琶洲互联网创新集聚区、金融城、珠江新城融合发展。

　　三是开展城乡环境综合治理。完善大气污染源清单，推进燃煤电厂环保升级改造，加快推进产业园区集中供热建设，提前实施机动车国Ⅴ排放标准，全面完成209家重点挥发性有机物企业污染治理，加强港口船舶尾气污染防治，加大对节能和新能源汽车发展的支持力度，推广清洁能源和可再生能源，增加管道燃气用户50万户，PM2.5年均浓度力争达到国家标准。全面开展流溪河流域水环境综合整治，加大城市黑臭水体整治力度，推进51条河涌整治工程，基本完成其中16条广佛跨界河涌治理，建设14座污水处理厂和57个农村污水治理设施。加快建设北江引水工程、北部水厂一期及牛路水库工程，完善农村自来水改造工程，城市集中式饮用水源水质达标率稳定在100%。推进生活垃圾源头减量、分类投放、分类收运，加快12个垃圾终端处理设施建设，推进农村生活垃圾治理，巩固生活垃圾分类示范城市创建成果。打造景观品质提升工程、森林生态工程和绿道、社区绿化等民生工程，推进海珠湿地等工程，创建全国绿化模范城市。划定永久基本农田和生态保护红线，探索建立生态补偿机制。

　　四是有序推进城市更新发展。强化土地节约集约利用，以功能区、轨道交通沿线等存量土地为重点，推动15平方公里土地储备。落实城市更新政策，完善制度体系。巩固提升同德围、金沙洲更新改造成果，加快推进罗冲围、大坦沙岛、广纸片区、鱼珠旧城等成片连片改造。推动中心城区旧厂房升级改造和旧社区、旧村庄微改造。盘活21宗“烂尾楼”，整治59条城中村。推动地下综合管廊建设，强化地下管线管理。健全城市能源供应保障体系，新建16座变电站。加快美丽乡村建设，建成71个市级、89个区级美丽乡村，加强农村环境治理，深化中心镇建设，创建第二轮名镇名村。

　　五是建设干净整洁平安有序城市。加强城市精细化管理，持续整治违法建设、“六乱”和违法户外广告，推进“五类车”综合治理。深化质量强市建设。开展“食品安全年”活动，创建国家食品安全城市，实施食品药品最严格全过程监管。落实安全生产责任制，加大监管执法力度，加强全民安全教育，及时排查治理事故隐患，预防和遏制重特大事故。健全应急体系和防灾减灾机制。深入开展群防群治，完善立体化社会治安防控体系和反恐机制，打击违法犯罪活动，推进监狱、看守所、戒毒所等政法设施建设。完善“五个一”平台综合信息系统。开展幸福社区提升计划，加强城乡基层协商民主建设，开展全国社区治理和服务创新试验区试点，实施社会组织品牌战略。加快现代社会组织体制建设，健全重大民生决策公众咨询监督及社会建设评价机制。实施“七五”普法，完善公共法律服务体系，健全社区矫正和人民调解机制，加大社会矛盾化解力度。加强来穗人员动态服务管理，开展重点地区和出租屋专项整治，创建来穗人员服务管理示范区。

　　（五）持续改善民生和发展社会事业。秉承坚持为民用心做事的理念，突出民生福祉，加强社会建设，推进基本公共服务均等化、优质化、便利化，让发展成果更多惠及市民群众。

　　一是办好十件民生实事。今年市本级投入50.8亿元，集中力量办好十件民生实事。1.加强食品安全监管；2.改善基层医疗卫生服务；3.促进创业就业；4.加大对困难弱势群体帮扶力度；5.缓解交通拥堵问题；6.推进垃圾处理设施建设；7.优化教育资源均衡配置；8.强化困难群体住房保障；9.提升公共安全保障；10.推动体育惠民。

　　二是完善社会保障体系。推进高校毕业生、城镇就业困难群体、农村劳动力就业创业帮扶。扩大五险一金覆盖面，提高企业退休人员、农转居人员和城乡居民养老金水平，稳妥推进机关事业单位养老保险制度改革，推进城乡居民医保扩面和异地就医即时结算，降低失业保险费率和工伤保险缴费标准，推行失业保险浮动费率，实施失业保险支持企业稳定岗位补贴，推进生育保险与医疗保险整合。健全最低生活保障与教育救助、医疗救助等衔接机制，建立临时救助制度，探索实施前置救助和支出型救助，加强流浪乞讨人员救助管理。推进社会福利、社会救助适度普惠，推动养老服务业综合改革试点，推进社区居家养老服务，加快养老机构建设，促进医养融合发展。

　　三是推进社会事业全面进步。发展教育事业，开展幼儿园规范化、义务教育民办学校标准化建设，加大中小学校建设力度，推动义务教育均衡优质发展，推进普通高中优质特色发展，落实15年免费特殊教育，加快广州教育城建设，加强职业技术人才培养，加强市属高校高水平大学建设，有序推进来穗人员随迁子女接受义务教育和高考，创建广东省推进教育现代化先进市。发展医疗卫生事业，建设医疗卫生高地，深化基层医疗卫生机构综合改革，推行家庭医生签约服务、基层首诊、双向转诊，加强住院医师和全科医生培训，提升妇产科、儿科的服务能力，做好传染性疾病防控和应急救治，稳步推进公立医院改革，鼓励社会资本举办医疗机构，落实“全面两孩”政策。做好迎接国家卫生城市复审工作。发展文化事业，加快28个重点文化设施建设，举办羊城国际粤剧节、中国（广州）纪录片节、国际版权博览会、纪念孙中山先生诞辰150周年等大型活动，开展农村电影放映、周末剧场演出等文化惠民活动，打造粤剧红船名片。发展体育事业，提升体育惠民服务水平，继续办好广州马拉松赛、国际龙舟邀请赛等体育品牌赛事活动，积极参与国内外竞技体育比赛。加强城市民族工作，促进宗教关系和谐。做好“双拥”优抚安置工作，推进军民融合发展，加快气象现代化，继续做好国防动员、海防、打私、打假、侨务、统计、对台、应急、地震、民防、参事、文史、文化遗产保护、档案、保密、修志、慈善等工作。

　　（六）建设服务型政府。坚持把加强政府自身建设作为增强政府执行力和公信力的突出任务来抓，不断提升政府治理能力和治理水平。

　　一是坚持依法行政。加强法治政府建设，依法设定、行使权力，强化权力制约和监督，落实重大行政决策合法性审查制度、重大决策终身责任追究制度及责任倒查机制，切实将政府活动全面纳入法治轨道。贯彻广州市法治政府建设规划要求，创建珠三角法治政府示范区。进一步简化优化公共服务事项，推进电子征信应用，坚决砍掉各类无谓的证明和繁琐的手续，提高行政执法能力，方便市民群众办事。依法及时向市人大及其常委会报告工作，认真执行市人大及其常委会各项决议和决定，切实做好人大代表建议和政协提案办理工作，提高质量和成效，自觉接受监督。

　　二是坚持政务公开。以公开为常态，不公开为例外，创新政务公开形式和机制，拓展政务公开内容，推进决策、执行、管理、服务和结果“五公开”。健全依法申请公开政府信息制度，全面公开公共服务事项，完善受理、审查、处理、答复等环节的工作程序，建设政府数据统一开放共享平台，加快推进党委政府、各区、各部门间信息共享和业务协同。继续推行重大行政决策公众参与、专家论证，定期组织政府新闻发布会。

　　三是坚持廉洁从政。要严守党的政治纪律和政治规矩，做政治上的明白人。持之以恒落实中央八项规定精神，改进政府机关作风，坚持暗访、查处、追责、曝光“四管齐下”，及时发现处置懒政怠政现象，严防“四风”反弹。加强行政监察，发挥审计监督作用，加大问责力度，对公共资金、公共资源、国有资产严加监管。建立预防腐败清单制度，开展巡察工作全覆盖，加强重大工程、重点项目和专项经费等领域的廉政风险防控，对苗头性问题及时约谈函询诫勉。持续保持惩治腐败高压态势，坚持无禁区、全覆盖、零容忍惩治腐败。

　　各位代表、同志们，实现今年和“十三五”时期发展目标，前景光明、任务繁重。让我们紧密团结在以习近平同志为总书记的党中央周围，在市委的坚强领导下，紧紧依靠全市人民，万众一心、积极作为，为巩固和提升国家中心城市地位、率先全面建成小康社会而努力奋斗！
